# URBAN OLDER ADULTS PERSPECTIVES ON NATURE: A DIRECTED CONTENT ANALYSIS

**DOI:** 10.1093/geroni/igad104.3030

**Published:** 2023-12-21

**Authors:** Jessica Cassidy, Kathy Lee

**Affiliations:** University of Texas at Arlington, Arlington, Texas, United States; University of Texas at Arlington, Arlington, Texas, United States

## Abstract

Biophilia is a theory which hypothesizes humans have an instinctual desire to interact with nature that in turn, supports health and well-being outcomes through gains in physical and mental functioning. This study aimed to explore how concepts outlined in the theory of Biophilia related to urban older adults’ experiences and perceptions of nature. This secondary data analysis examined five focus groups with community-dwelling, urban older adults. All participants were female, and majority were African Americans (83.3%). Using directed content analysis, we developed a codebook from concepts and mechanisms outlined in the theory of Biophilia. Our analysis resulted in six themes, 1) Infinite wonder of nature; 2) Nature activating long-term memories; 3) Nature influencing feelings of well-being; 4) Connecting with nature; 5) Conservation ethic; and 6) Nature across the lifespan. Overall, the data fit the theory well. Through the lens of Biophilia we understood why the sample of participants valued their interactions with nature, as well as how experiences in nature promoted their feelings of well-being. We observed appreciation for nature interactions can deepen in later life suggesting nature-based interventions may be especially well-suited for older adult populations. We suggest the theory of Biophilia can lend well to public health approaches towards addressing the social and economic implications presented by growing numbers of urban dwelling older adults. Moreover, Biophilia can provide a theoretical basis for tailoring nature-based interventions delivered across a variety of settings to match diverse study goals and overall, to promote the health and well-being of older adults.

